# Investigating the mechanisms of peritoneal metastasis in gastric adenocarcinoma using a novel ex vivo peritoneal explant model

**DOI:** 10.1038/s41598-022-13948-x

**Published:** 2022-07-07

**Authors:** Deanna Ng, Aiman Ali, Kiera Lee, Denise Eymael, Kento Abe, Shelly Luu, Karineh Kazazian, Yi Qing Lu, Savtaj Brar, James Conner, Marco Magalhaes, Carol J. Swallow

**Affiliations:** 1 Institute of Medical Science, University of Toronto, Toronto, Canada; 2 Lunenfeld-Tanenbaum Research Institute, Sinai Health System, Toronto, Canada; 3 Faculty of Dentistry, University of Toronto, Toronto, Canada; 4 Department of Surgical Oncology and Division of General Surgery, Princess Margaret Cancer Centre, University Health Network/Mount Sinai Hospital, 600 University Avenue #1225, Toronto, ON M5G 1X5 Canada; 5 Department of Surgery, University of Toronto, Toronto, Canada; 6 Department of Pathology and Laboratory Medicine, Mount Sinai Hospital, Toronto, Canada

**Keywords:** Biological models, Cancer models, Gastrointestinal cancer, Metastasis

## Abstract

Gastric adenocarcinoma, commonly known as stomach cancer, has a predilection for metastasis to the peritoneum, which portends limited survival. The peritoneal metastatic cascade remains poorly understood, and existing models fail to recapitulate key elements of the interaction between cancer cells and the peritoneal layer. To explore the underlying cellular and molecular mechanisms of peritoneal metastasis, we developed an ex vivo human peritoneal explant model. Fresh peritoneal tissue samples were suspended, mesothelial layer down but without direct contact, above a monolayer of red-fluorescent dye stained AGS human gastric adenocarcinoma cells for 24 h, then washed thoroughly. Implantation of AGS cells within the explanted peritoneum and invasion beyond the mesothelial layer were examined serially using real-time confocal fluorescence microscopy. Histoarchitecture of the explanted peritoneum was preserved over 5 days ex vivo. Both implantation and invasion were suppressed by restoration of functional E-cadherin through stable transfection of AGS cells, demonstrating sensitivity of the model to molecular manipulation. Thus, our ex vivo human peritoneal explant model permits meaningful investigation of the pathways and mechanism that contribute to peritoneal metastasis. The model will facilitate screening of new therapies that target peritoneal dissemination of gastric, ovarian and colorectal cancer.

## Introduction

The peritoneal cavity is a potential space that is enveloped in a thin layer of specialized tissue known as the peritoneum^1,2^. The peritoneal surface can be the target of a variety of inflammatory, infectious, and neoplastic processes, such as peritonitis associated with perforated appendicitis, tuberculosis and peritoneal carcinomatosis, respectively^3,4^. The predilection of certain types of primary cancer to spread within the peritoneum has long been the subject of scientific inquiry^5^. The peritoneal niche is considered a “soil” within which certain epithelial malignancies characteristically “seed”. Prominent examples include serous ovarian carcinoma, gallbladder and gastric adenocarcinomas^6,7^. The cellular and molecular features that enable this specialized interaction remain largely undefined.

In the case of gastric adenocarcinoma (i.e. stomach cancer), metastasis to the peritoneum remains a common and challenging problem. In North America and Europe, approximately 35% of patients who are newly diagnosed with gastric cancer already have peritoneal metastases, and the median survival in such patients is ~ 4 months^8–10^. Of those patients without overt distant metastases at presentation who then undergo curative intent resection, about one quarter develop peritoneal recurrence, with a median survival of 6 months even when treated with cytotoxic chemotherapy^11,12^. Peritoneal metastases from gastric adenocarcinoma can cause ascites, ureteric and bowel obstruction, and intractable symptoms that are difficult to manage effectively^13,14^. Peritoneal carcinomatosis degrades both quantity and quality of life. Identification of the cellular mechanisms and distinct molecular profiles that predispose to peritoneal spread will lead to discovery of rational targets for the prevention and/or treatment of this stubborn metastatic pattern.

**Figure 1:**
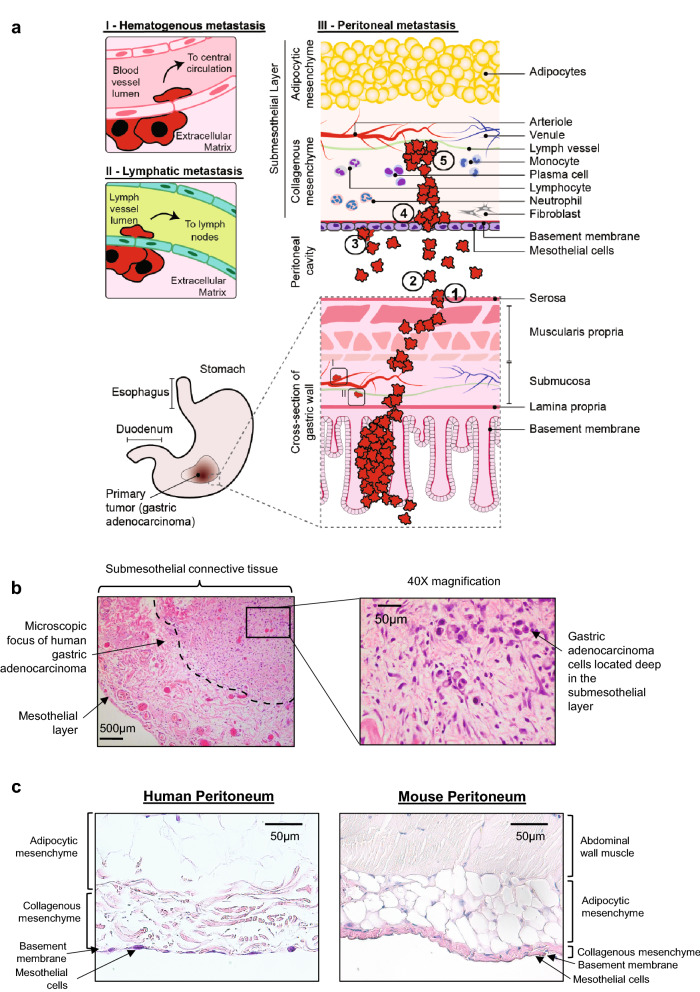
Human peritoneum represents a distinct niche for dissemination of gastric adenocarcinoma. (**a**) Cartoon depicting routes of metastatic spread from the primary tumour in gastric adenocarcinoma. The hematogenous (**I**) and lymphatic (**II**) metastatic cascades involve directional intra- and extravasation of cancer cells in and out of vessels. This is not the case for the peritoneal metastatic cascade (**III**), which comprises 5 major steps: (1) detachment of cancer cells from the primary tumour at the serosal surface of the stomach; (2) survival and movement within the microenvironment of the peritoneal cavity; (3) attachment of free tumour cells to peritoneal mesothelial cells; (4) invasion into the submesothelial layer and (5) proliferation of cancer cells within the submesothelial mesenchyme. (**b**) Metastatic deposit within the peritoneum. Cross sectional image taken perpendicular to mesothelial surface showing established gastric adenocarcinoma peritoneal metastasis deep in submesothelial layer, with surrounding fibrotic response (Dashed line depicts periphery of peritoneal metastasis). (**c**) Representative images of formalin-fixed paraffin-embedded (FFPE) sections of human (left) and mouse (right) peritoneum stained using H&E (10X magnification) illustrating significant differences in histoarchitecture, including a thinner layer of collagenous mesenchyme in the mouse.

However, elucidation and validation of the mechanisms of peritoneal metastasis have proven difficult. Peritoneal dissemination of cancer is a complex and dynamic process that, like the hematogenous metastatic cascade, can be described as a series of discrete steps. The peritoneal metastatic cascade is conceptualized to comprise 5 main steps (Fig. 1a): (1) detachment of cancer cells from the primary tumor and penetration through the serosa; (2) survival in the microenvironment of the peritoneal cavity; (3) attachment of cancer cells to peritoneal mesothelial cell layer; (4) invasion into the submesothelial space; and (5) growth within the peritoneum as a metastatic deposit^15–17^. The peritoneal metastatic cascade notably differs from the hematogenous and lymphatic cascades, both of which entail directional, intra- and extra-vasation through vessel walls (Fig. 1a)^18–20^. The study of an established gastric cancer peritoneal metastasis, such as that shown in Fig. 1b, can be hampered by cancer cell-induced fibrosis, and admixture with inflammatory cells. In addition, the molecular features that enabled steps 1 through 4 of the peritoneal metastatic cascade may no longer be relevant/represented.

Our current understanding of the mechanisms of peritoneal carcinomatosis in gastric adenocarcinoma has been formulated based on artificial in vitro cellular representations of the human peritoneum (e.g. a monolayer of mesothelial cells plated on type IV collagen matrix), and on animal models^21–23^, most of which do not recapitulate the entire metastatic cascade, and have the added disadvantage that they employ immunodeficient animals^24–26^. The marked histoarchitectural differences between mouse peritoneum and human peritoneum are clear upon routine histologic assessment (Fig. 1c). In vitro model systems do not facilitate study of complex cellular behaviours within a 3D environment such as the peritoneal cavity and peritoneum^27–29^.

We aimed to create a more relevant model of the human peritoneal metastatic cascade, by allowing gastric cancer cells to interact with fresh explanted human peritoneum ex vivo. We used the human gastric cancer line AGS, which lacks E-Cadherin (CDH1) and in this regard, models diffuse type gastric adenocarcinoma, which has a strong predilection for peritoneal spread^30,31^. Having demonstrated the viability, specificity and reproducibility of the explant model in measuring implantation and invasion of human gastric cancer cells into human peritoneum, we validated the model through restoration of functional CDH1, demonstrating its applicability as an investigational tool.

## Results

### Establishment of ex vivo peritoneal metastasis model

**Figure 2:**
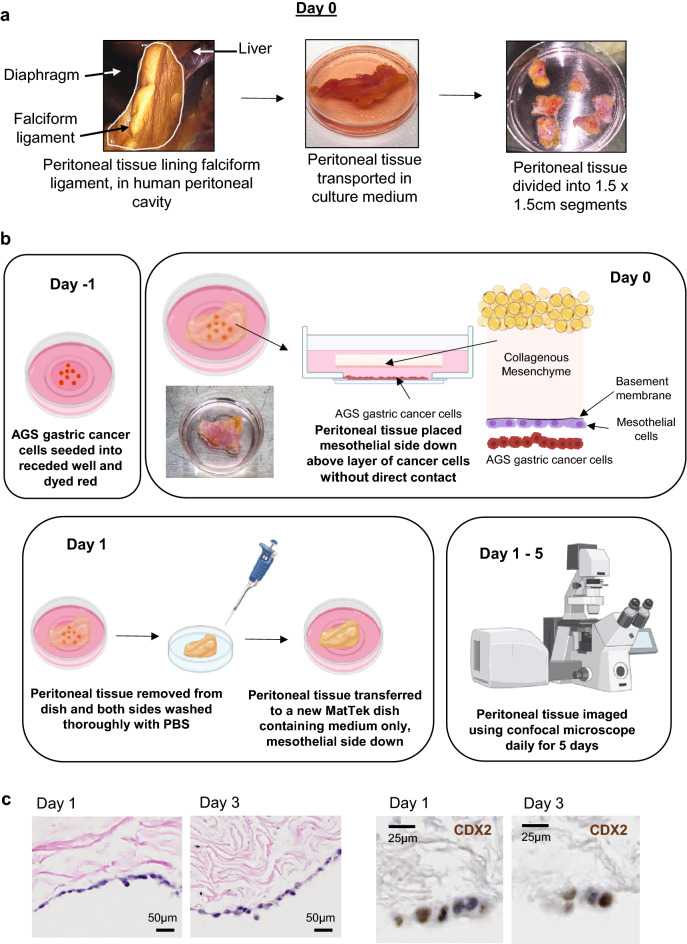
Ex vivo human peritoneal metastasis model: experimental set up and timeline. (**a**) On day 0, a sample of peritoneal tissue from a patient undergoing elective laparotomy or laparoscopy in the operating room is harvested, transported to the laboratory in sterile culture medium, and divided into smaller segments. (**b**) Experimental workflow. Day -1: AGS gastric cancer cells are seeded into receded well of MatTek dish and dyed red using Cell Tracker cm-DiI (Thermofisher). Day 0: Each segment of human peritoneal tissue is placed mesothelial side down floating in medium over a receded well, with no direct contact between the cells and the tissue. Day 1: Peritoneal tissue is removed from dish and washed thoroughly with PBS before transfer into a fresh MatTek dish with medium only, mesothelial side down. Images were taken with a confocal microscope daily from Day 1 to 5. In parallel, peritoneal tissue samples were subjected to tissue processing (FFPE) daily (see Fig. S1). (**c**) Representative images of FFPE sections of ex vivo peritoneum at Days 1 and 3, stained with hematoxylin and eosin (H&E) (20X magnification) (left) or stained by immunochemistry for CDX2. AGS cells along the mesothelial surface stain positive for CDX2, a marker of adenocarcinomas derived from an upper gastrointestinal source.

Fresh human peritoneal tissue samples were obtained from patients undergoing elective abdominal surgery at Mount Sinai Hospital, Toronto, with REB approval and informed patient consent (Fig. 2a, left panel; Supplemental Table 1). All methods were performed in accordance with the relevant guidelines and regulations. On the day prior to surgery (Day-1, Fig. 2b), 1 × 10^5^ AGS gastric cancer cells that had been dyed red with CellTracker™ CM-DiI were seeded into the 1.4 cm diameter receded well with an underlying glass window at the center of a 3.5 cm MatTek dish, then cultured overnight in RPMI/10% FBS (medium). On the day of surgery (Day 0), peritoneal tissue was transferred directly from the operative field to a Corning 100 mm Tissue Culture-treated culture dish filled with medium (Fig. 2a, right panels). Under sterile conditions in the laboratory, peritoneal tissue was divided into 1.5 cm^2^ segments, each of which was placed mesothelial side down into a pre-seeded MatTek dish, where it floated over the receded well, without direct contact between the cancer cells and the peritoneal tissue (Fig. 2b top, right panel; Fig. S1a). The dish was then incubated at 37 °C for 24 h, following which the peritoneal tissue was removed and placed in a fresh 10 cm Petri dish, then washed vigorously on both sides with PBS before being transferred to a new MatTek dish containing medium only, again mesothelial side down (Fig. 2b, bottom left panel). The MatTek dish allows for live cell imaging from below through the glass window, using confocal microscopy. Fluorescence images were obtained with a LEICA SP8 confocal microscope, daily for 5 days (Fig. 2b, bottom right panel).

To confirm that the co-cultured explants remained intact and viable over the planned course of the experiment, parallel samples were subjected to conventional histologic examination. Over serial daily intervals, the peritoneal tissue was removed from the MatTek well and processed *in toto*^32^*.* The entire tissue block was fixed in 10% formaldehyde for 24 h, then sliced into into thin strips (50 mm) at right angles to the peritoneal surface (mesothelial layer, colored beige in Fig. S1b). The entire block was cut at 2 mm intervals, yielding twenty 5 µm strips (Fig. S1c,d). This ensured that the entire strip of peritoneal tissue was sampled. Stained slides were evaluated by a specialized GI pathologist (J.C.), blinded to the experimental group. Examination of H&E stained sections revealed viable appearing cells of two morphologic types along the mesothelial surface of the explanted peritoneum at days 1 and 3 (Fig. 2c, left panels): thin, flat cells were consistent with mesothelial cells while plump, round cells with prominent nuclei were AGS cells. This was confirmed by immunohistochemical staining for the gastrointestinal epithelial marker CDX2 which stained AGS cells, as shown on days 1 and 3 (Fig. 2c, right panels).

### Cyto-architecture of explanted human peritoneal tissue is preserved ex vivo

**Figure 3:**
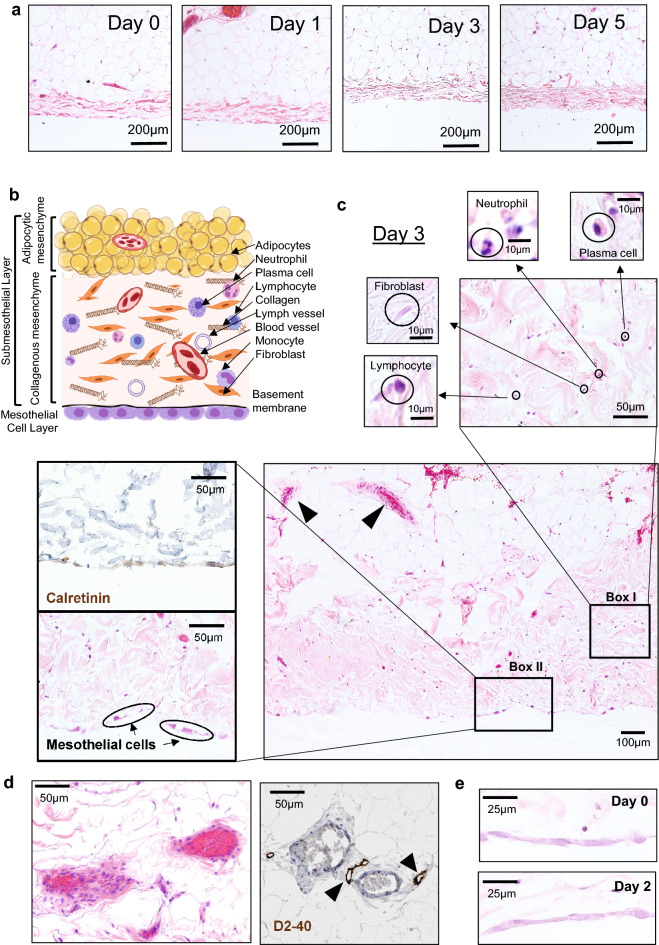
Cyto-architecture of human peritoneum is preserved ex vivo*.* (**a**) Representative images of sections of freshly explanted human peritoneum (Day 0) and on Days 1, 3 and 5 following harvest, processed as described (see “Methods” section and Fig. S1), stained using H&E (X10). Preservation of cyto-architecture over the experimental timeframe is demonstrated. (**b**) Cartoon illustrating the cellular and stromal components of normal human peritoneum, consisting of a mesothelial layer (mesothelial cells and underlying basement membrane) and a complex submesothelial layer, which is made up of a largely collagenous mesenchyme and an underlying adipocytic mesenchyme. (**c**) On Day 3 post explant, intact blood vessels are observed in the submesothelial layer (arrowheads, bottom right panel). Fibroblasts, neutrophils, lymphocytes and plasma cells are present in the collagenous mesenchyme (Box I). Mesothelial cells stain positive for calretinin (Box II). (**d**) Blood vessels in the submesothelial layer are accompanied by lymphatic vessels, which stain positive for D2-40 (arrowheads). (**e**) Representative images of sections of human peritoneal tissue stained using H&E (20X) showing intact mesothelial cells at Days 0 (top) and 2 (bottom).

Explanted tissues have a variably limited life span ex vivo. Resident cells of the peritoneum inhabit a specific microenvironmental niche that exposes them to peritoneal fluid, which is characterized as relatively hypoxic, hypoglycemic, and hypercarbic^33^. To determine how the ex vivo culture conditions used in our model affected the integrity of human peritoneum, we analyzed its structure over the planned experimental time course. Examination of H&E stained sections showed that the cyto-architecture of the peritoneal samples appeared to remain essentially intact over a period of 5 days ex vivo (Fig. 3a). The cellular composition of the explanted peritoneum largely mirrored that expected for fresh human peritoneum (Fig. 3b,c)^2^. The submesothelial adipocytes retained their distinct appearance (Fig. 3a), and apparently viable fibroblasts and leukocytes were also observed in the submesothelial layer, as shown on Day 3 (Fig. 3c, large panel). Additional evidence for the viability of leukocytes resident within the peritoneal tissue was obtained by profiling the cytokines present in the culture medium conditioned by the explants (Supplemental Table 2). As shown in Fig. 3d, intact microvascular and lymphatic structures were observed in the adipocytic layer on Day 3. Delicate mesothelial cells line the peritoneal surface, and are supported on a basement membrane composed of type IV collagen and laminin^1^. An intact layer of mesothelial cells was observed on Days 0 through 2 after peritoneal explantation (Fig. 3e). By Day 3, the mesothelial layer had become more patchy (see staining for calretinin on Day 3, Fig. 3c) and was lost by Day 5 (Fig. 3a). This is consistent with the weak attachment of the mesothelial cells to the basement membrane observed in vivo^3^, potentially exaggerated by the ex vivo culture conditions.

### Assessment of interaction between cancer cells and explanted peritoneum

**Figure 4:**
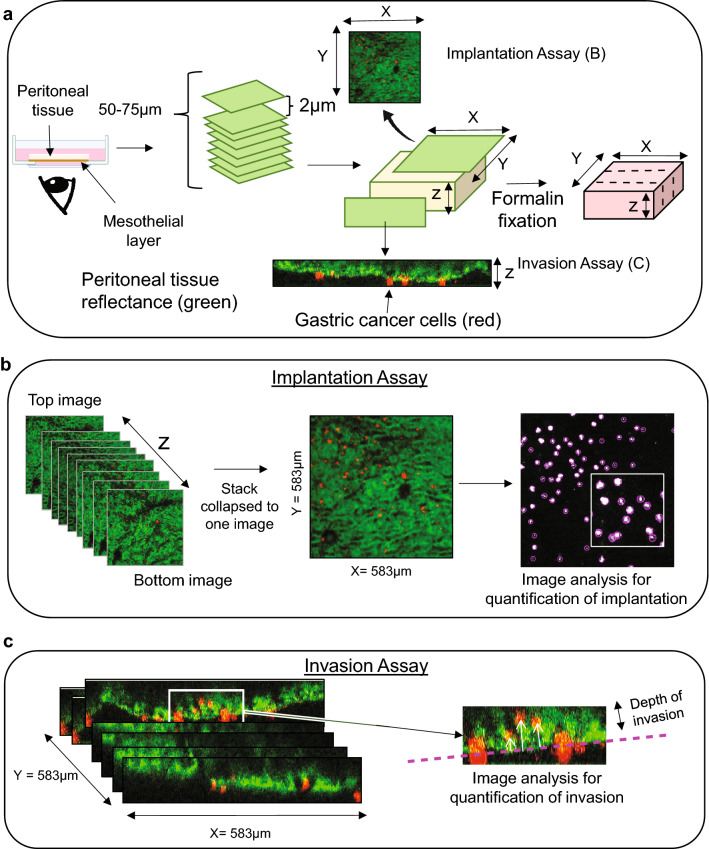
Methods used to assess implantation and invasion of cancer cells into human peritoneal tissue. (**a**) Peritoneal tissue was imaged using confocal microscopy and a stack of images, 2 µm apart, was generated (total depth 50–75 µm). Background peritoneal tissue appears green (reflectance of collagen fibers), and cancer cells appear red (staining with Cell Tracker cm-DiI). (**b**) To assess cancer cell implantation, the Z-stack of images was collapsed into one image and the number of cells within the peritoneal tissue was measured by selecting the red channel only. The number of cells was determined using FIJI plugin TrackMate (see “Results” section for detailed calculations). (**c**) To assess depth of cancer cell invasion into peritoneal tissue, distance between the mesothelial cell layer (fuchsia dashed line) and the mid-point of the cell was measured on orthogonal view (FIJI); the three cells that had invaded the mostly deeply across all of the orthogonal views generated for each 583 × 583 µm square were identified, and their mean depth of invasion calculated (see “Results” section).

As illustrated in Fig. 2b, AGS cancer cells were allowed 24 h to interact with the explanted peritoneal tissue suspended over them, following which the peritoneal sample was removed and washed thoroughly to eliminate non-adherent cells. AGS cells that remained “implanted” within the peritoneal tissue were then imaged serially in two separate assays: an Implantation Assay and an Invasion Assay (Fig. 4a–c). For both assays, the peritoneal tissue was imaged from beneath the mesothelial surface using a confocal microscope to capture a stack of images 2 µm apart, for a total depth of 50–75 µm (z axis). The AGS cancer cells, stained red with with CellTracker™ CM-DiI, appear distinct against the background green reflectance of the peritoneal tissue’s collagen fibres. For each 1.5 × 1.5 cm peritoneal sample and experimental condition, five random unique areas of 583 X 583 µm were assessed daily. For the Implantation Assay (Fig. 4a top, b), the entire z stack of images was collapsed into one *en face* image, and the number of AGS cells associated with the peritoneal tissue was measured using FIJI plugin Track Mate^23^, selecting the red channel only. The average number of AGS cells present in each 583 × 583 µm square of the peritoneal tissue was calculated based on image analysis of 5 representative squares (Fig. 4b shows a representative image from Day 2).

For the Invasion Assay (Fig. 4a bottom, c), depth of cancer cell invasion into the peritoneal tissue was measured serially over 5 days. The acquired stack of confocal images was reconstructed into a 3D structure that was then analyzed as 512 slices 1.14 µm apart (Y axis). The 512 slices were scanned for the position of the AGS cells relative to the mesothelial surface, and depth of invasion for an individual cell was defined as the distance from the mesothelial surface (shown in fuchsia in Fig. 4c) to the mid-point of the cell (illustrated by tip of white arrows in Fig. 4c) in an orthogonal view. The average depth of the three deepest cells was recorded after scrolling through all of the orthogonal views (> 500) generated for each of 5 representative 583 × 583 µm squares, and the Invasion score recorded for a given peritoneal sample was the mean of the 5 values (Fig. 4c shows an example from Day 5). This calculation was done manually by two independent observers and the scores were combined and averaged to yield a final Invasion score for each peritoneal sample, for each day.

### Ex vivo model is sensitive to gene manipulation

**Figure 5:**
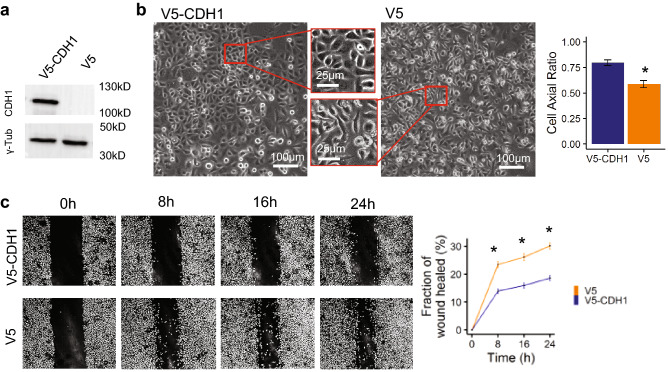
Restoration of functional E-cadherin (CDH1) restores epithelial phenotype and suppresses migration in AGS gastric cancer cells. (**a**) Extracts from AGS cells expressing V5-tagged CDH1 or V5 alone were subjected to immunoblot, using anti-CDH1 antibody. γ-Tubulin is probed as a control. (**b**) Brightfield images showing that restoration of functional CDH1 in AGS cells resulted in a more epithelial phenotype (left) compared to V5 tagged AGS cells. Quantification of cell shape where 1 represents a perfect circle shows that cell axial ratio is increased (rounder) in AGS cells expressing functional V5-CDH1 versus V5 alone. (**c**) Directional migration, assessed in a wound healing assay, is reduced in AGS cells expressing functional V5-CDH1 versus V5 alone, as seen in serial brightfield images (left panels), with quantification shown in right panel. Data are presented as mean ± SEM of 3 independent experiments, * = *p* < 0.01 versus V5-CDH1.

To determine whether the explant model would permit detection of alterations in peritoneal implantation and invasion by genetically modified AGS cells, we reprogrammed the latter to restore an epithelial phenotype. Parental AGS cells do not express functional CDH1 due to a truncation mutation (T578fs)^34^. Using lentiviral infection of AGS cells, we stably restored wild-type CDH1 (Fig. 5a, S2a), and observed the expected reversal of the more mesenchymal phenotype seen in V5-alone controls (Fig. 5b). Restoration of functional CDH1 also suppressed 2D migration (Fig. 5c), without altering AGS cell viability or proliferation (Fig. S2b,c). Though plating density was equivalent (Fig. S2d, left panels), we noted an increase in confluence by Day 1 for CDH1 + cells (Fig. S2d, right panels). Indeed, measurement of the number of AGS cells in suspension in the culture medium just before removal of the explant showed a significant reduction for V5-CDH1 vs. V5 alone (Fig. S2e).

**Figure 6:**
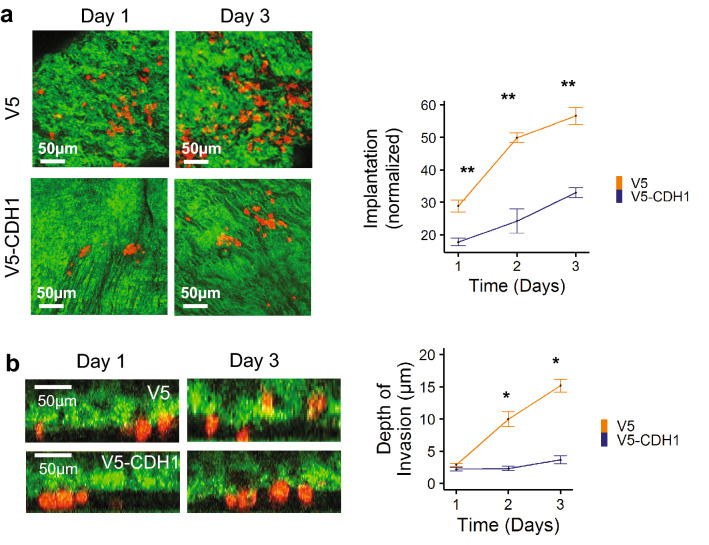
Restoration of functional E-cadherin (CDH1) suppresses implantation and invasion into the peritoneum by AGS cancer cells. (**a**) En-face fluorescence time-lapse images (left) of ex vivo peritoneal tissue showing presence of AGS cells expressing V5-CDH1 or V5 alone implanted within the peritoneum (implantation). Implantation was quantified by measuring the number of cells present in one 583 × 583 µm square multiplied by 662 such squares per explant, normalized according to the number of live cells available for implantation under each condition (see “Results” section for detailed calculations). V5-CDH1 expressing cells showed a marked reduction in implantation compared to V5 alone controls (right). (**b**) Cross sectional fluorescence time-lapse images (left) of ex vivo peritoneal tissue showing depth of invasion by AGS cells expressing V5-CDH1 or V5 alone. Quantification showed reduced invasion by AGS cells with V5-CDH1 expression (right). Data are presented as mean ± SEM of 3 independent experiments, *p < 0.01, **p < 0.001 versus V5-CDH1.

We next examined the effect of CDH1 restoration on the ability of AGS cells to implant and invade into fresh explanted human peritoneum. To account for the limiting effect of CDH1 rescue on the number of live cells in suspension available to implant into the explant, Implantation was calculated as the average number of cells present in one 583 × 583 µm square multiplied by 662 such squares per explant, divided by the number of live AGS cells present in suspension on day 1, before the explant was washed and removed from the initial MatTek dish. CDH1 rescue dramatically suppressed both implantation (Fig. 6a) and invasion (Fig. 6b) of AGS cells into the explanted peritoneum. This result confirmed the sensitivity of the model to manipulation of cancer cell genotype/phenotype, demonstrating its relevance in studying the molecular determinants of peritoneal metastasis. The sequential increase in the number of cells observed within the peritoneum between days 1 and 3 indicates that the implanted AGS cells were proliferating in this microenvironment.

### Human cell lines screened for implantation in peritoneal explant model

We further tested a variety of human cell lines for peritoneal implantation in our model (Fig. S3a,b). The non-malignant line RPE did not detectably implant, nor did the soft tissue sarcoma line HT1080 or the osteosarcoma line U2OS. Carcinoma lines derived from lung (adenocarcinoma AC549), oropharynx (squamous cell carcinoma UMSCC1) and urinary bladder (transitional cell carcinoma T24) also failed to implant. By contrast, cell lines derived from cancer types that are known to metastasize to the peritoneal cavity in patients, including adenocarcinoma of the ovary (HEY), cervix (HeLa), pancreas (Panc1) and colorectum (Hct116) did implant within human peritoneal explants. Thus, the model appeared to reflect predisposition to peritoneal dissemination in patients.

## Discussion

Here, we have established an ex vivo co-culture system that uses freshly harvested human peritoneal samples as a tool to investigate interaction of gastric cancer cells with the peritoneum, and penetration through the mesothelial layer into the submesothelial space. This system models initial steps of the peritoneal metastatic cascade, overcoming several limitations of previously described in vitro and in vivo model systems. The cytoarchitecture of the explanted peritoneal tissue is retained, recapitulating both the physical and cellular barriers to implantation and invasion present in vivo. The model allows investigation of gene expression profiles that enable or suppress peritoneal metastasis, as demonstrated by restoration of wild type CDH1 gene product in AGS cells with resultant disabling of peritoneal implantation and invasion.

As far as we are aware, only one other ex vivo human peritoneal model that investigates gastric cancer dissemination has been published to date. In that model, which used peritoneum removed as part of a hernia sac during elective hernia repair, gastric cancer cells were seeded densely directly onto the peritoneum^35^. By contrast, in the model we developed and describe here, gastric cancer cells are not placed in physical contact with the peritoneum, since they are cultured in a monolayer at the bottom of a receded well, at a distance of 750 µm from the peritoneal mesothelium. For peritoneal implantation to occur, this platform requires active movement of cells within a liquid microenvironment and against the force of gravity, mimicking the in vivo microenvironment. Other distinguishing features of our model are the histologic verification of maintenance of normal peritoneal cytoarchitecture, and real time serial fluorescence imaging of the cancer cells as they move into and within the peritoneal surface. The cell tracker dye becomes membrane impermeant once it is within the cell, and continues to fluoresce over 3–6 subsequent cell divisions, meaning that the cell and its progeny can be followed over time^36^. The peritoneal tissue is visualized through its endogenous reflectance, so that fixation and staining are not required^37^. The automated quantification of cellular implantation using FIJI trackmate eliminated observer bias and inter-observer variability^38^.

Having established the sensitivity and discriminatory capabilities of the ex vivo model described here, we anticipate various extensions of the experimental design that would enable more detailed investigation of several aspects of the peritoneal metastatic cascade. Initial attachment/adhesion to the peritoneal mesothelium and interaction with the mesothelial cells could be studied within the first few hours of co-culture. Cancer cells that implant may be compared to those that do not to screen for enabling expression profiles in an unbiased manner. Drugs and other compounds with the potential to target specific aspects of the peritoneal metastatic cascade can be screened^39^ (see Fig. S4 as an example), and deleterious side effects on the peritoneal tissue itself potentially detected.

We had anticipated challenges with reproducibility of results, given that the peritoneal tissue samples we used came from a variety of patients with a range of comorbidities, who were undergoing abdominal surgery for different pathologies. Though the median age of these patients was 61, it ranged from 28 to 80 (n = 9, Supplemental Table 1). While there may have been inter-patient variability in terms of submesothelial stroma thickness and composition^3^, the depth of penetration of the cancer cells over the time line studied was rather limited, and this favoured reproducibility of results. All of the included patients were undergoing resection of an intra-abdominal primary cancer, in the absence of any detectable metastases. In particular, there were no cases with any gross abnormality of the peritoneum, making it unlikely that peritoneal mesothelial cells had already undergone mesenchymal transition at the time of explantation, but this was not specifically ruled out in the present experiments.

The model we describe here is not conducive to study of the effects of cancer cells on peritoneal mesothelial cells, given the disappearance of the latter from the surface of the explants by day 5. In addition, the relatively short time frame of the interaction between cancer cells and the explants (24 h) was unlikely to be sufficient to observe conversion of mesothelial cells into carcinoma-associated fibroblasts (CAFs) via mesothelial-to-mesenchymal transition (MMT)^40^. The mesothelial metastatic niche represents an important influence on the peritoneal metastatic cascade^41^, and should be considered in evaluating any peritoneal-directed therapies. This is a limitation of the explant model we describe here. Nevertheless, the present model captures other elements of the early phases of peritoneal metastasis, before the implanted cells proliferate into a colony, acquire a blood supply and engender a fibrotic response, as seen in well-established peritoneal metastases (e.g. Fig. 1b)^42^. The main advantage of the explant model is the ability to focus on these initial processes and what enables them. In terms of implications for discovery science and clinical translation, this ex vivo peritoneal metastasis model offers the capacity to screen genes/pathways of interest, as well as novel therapies in a relatively simple co-culture system that recapitulates key aspects of the in vivo microenvironment. It is also potentially applicable to other cancer types that harbour a predisposition to peritoneal dissemination, such as ovarian, pancreatic and colon cancers^5,43^, though formal validation should be performed for each cancer cell type.

Overall, this model permits increased understanding of the mechanisms and drivers of peritoneal metastasis. Therapies targeted to these enabling pathways have the potential to improve quality of life and survival in patients suffering from advanced gastric cancer, and other malignancies that hone to the peritoneum.

## Methods

### Cell culture

Cells were grown at 37 °C, 5% CO_2_ in DMEM (HeLa, Hct116, HEY, T24), RPMI1640 (AGS, Ht1080, Panc1, RPE) or McCoy 5A medium (USMCC1, U2OS) supplemented with 10% Fetal Bovine Serum (FBS) (Wisent). AGS, USMCC1, HEY, HT1080, and HeLa cell lines were obtained from ATCC in 2018, 2016, 2013, 2010 and 2009, respectively, and were maintained between passages 4 and 15. The U2OS line was a kind gift from the Laurence Pelletier laboratory, Lunenfeld Tanenbaum Research Institute, Sinai Health System, Toronto, Canada (obtained in 2013 and maintained between passages 6 and 10). Panc1, Hct116, T24 and RPE lines were a kind gift from the Daniel Schramek laboratory, Lunenfeld Tanenbaum Research Institute, Sinai Health System, Toronto, Canada (obtained in 2020 and maintained between passages 8 and 28). Cell lines were not further authenticated in our laboratory. For drug treatment experiments, AGS cells were exposed to centrinone B (5690, TOCRIS Bioscience) dissolved in DMSO, at a final concentration of 500 nmol/L in co-culture medium, 24 h before peritoneal tissue was placed into the MatTek dish.

### Creation of stable wildtype CDH1 expressing cell lines

AGS cells, which are derived from primary gastric adenocarcinoma, do not express CDH1 protein due to a truncating mutation (T578fs)^34^. The parental AGS cell line was infected with lentivirus to stably express V5-CDH1, or V5 alone, using gateway constructs (pLEX_306m, Addgene). Lentiviruses were produced as described^40^ and used to infect the cells for 24 h, followed by puromycin (2ug/ml) selection for 7 days. To confirm protein expression, cell extracts were separated by SDS-PAGE, transferred onto PVDF membranes, blocked with 5% milk-PBS-0.1%Tween, probed with antibody to CDH1 (BD Transduction Laboratories, 610182, 1:1000) at 4 °C overnight, then HRP-linked secondary antibody (GE Healthcare), and detected using SuperSignal West Femto Maximum Sensitivity Substrate (34095; Thermo Fisher Scientific).

### Fluorescence-activated cell sorting

On Day 1, 24 h after exposing AGS cells to peritoneal tissue in a MatTek dish, the culture medium was collected and suspended cells treated with RBC lysis buffer (NH4Cl (155 mM), KHCO3 (10 mM), EDTA (0.1 mM) pH 7.6). Following clearance and resuspension in flow cytometry sorting buffer (Hanks Balanced Salt Solution, 25 mM HEPES pH 7.0, 2 mM EDTA, 1% Fetal Bovine Serum) with 4′,6-diamidino-2-phenylindole (DAPI, 0.2 uM) at a concentration of 5 X10^6^ cells per ml, flow cytometry-based counting, with gating settings that separated out non-AGS cells, and live-dead assay were performed. AGS cells that remained adherent to the receded well were harvested by trypsinization and similarly processed for counting and live-dead analysis. Samples were filtered through a 40 μm nylon mesh to eliminate large aggregates. The stained samples were immediately analyzed on a Fortessa 20 flow cytometer. Data were processed with FlowJo software.

### Histologic and Immunohistochemical (IHC) evaluation

All protocols were approved by the Sinai Health System Research Ethics Boards***. ***Tissue samples were fixed in 10% formalin for 24 h at room temperature. Serial sectioning was performed at three levels, 50 mm apart. Each tissue edge was stained red with Tissue-Marking dye to orientate the specimen for paraffin embedding (Fig. S1a). The strips of peritoneal tissue were paraffin embedded by lining up the three strips parallel to each other, with the mesothelial layer oriented to one side (Fig. S1b). Sections (5 μm) were stained with hematoxylin and eosin (H&E) according to standard protocols using the Veristain Gemini Automated Slide Stainer (Thermo Scientific) at the University of Toronto Dentistry Pathology Core, and examined with a Leica DMR upright microscope. For IHC staining, slides were stained with CDX2 1:50 or D2-40 1:50 antibodies using the fully automated Dako Omnis platform (deparaffinization and retrieval built in), using the Dako OMNIS detection kit (Ref: GV800). Slides were exposed to high pH for 15 min, antibody incubation for 10 min, polymer detection for 20 min, substrate chromogen for 5 min and finally Dako hematoxylin for 3 min. This IHC processing was performed at the Department of Pathology at the Hospital for Sick Children, Toronto.

### Cell staining for live cell imaging

AGS cells plated on MatTek dishes were stained using CellTracker™ CM-DiI (ThermoFisher, C7000). AGS cells were first plated on MatTek dishes for 18 h prior to staining. Media was removed from cells and cells were incubated with dye (1 μg/μl) for 30 min.

### Assessment of cell morphology

Images of cells grown to ~ 80% confluence on the 96-well plates were captured by INCell Analyzer 2000. FIJI was used to analyze the images and determine cell axial ratio (width/length). 30 × 10^5^ V5 or V5-CDH1 expressing AGS cells were imaged per well, 20 fields per well.

### Scratch wound migration

1 × 10^6^ cells were seeded into 6 well plates and serum starved for 18 h prior to scratch. Scratches were made manually using a P10 pipette tip, and migration was assayed for 24 h using the INCell6000 analyzer equipped with a motorized stage and a live cell apparatus (37 °C heated and humidified chamber with 5% CO_2_) with a 6-well plate adaptor. Data acquisition was performed continuously over the indicated time courses. Images were collected with a 10X objective lens. All hardware and image capture conditions were made possible, and images analyzed, using MATLAB 9.4 2018.

Image analysis was carried out by measuring the total wound area in three fields per condition, at t = 0, 8, 16 and 24 h. The residual wound area was expressed as a percent of original wound, which was itself highly reproducible, subtracted from 100 to yield the %healed at each time point, and compared across groups by t-test.

### Measurement of cell viability and proliferation

V5 or V5-CDH1 expressing AGS cells were seeded on a clear flat bottom 96-well black polystyrene plate (Corning), and cultured in RPMI medium supplemented with 10% FBS at 37 °C, 5% CO_2_. Cell viability (1 × 10^5^ cells plated) and proliferation (5 × 10^4^ cells plated) assays were performed at 24 h and 48 h. Cell viability was assessed using the LIVE/DEAD® Viability/Cytotoxicity Kit (ThermoFisher), according to the manufacturer’s instructions. For cell proliferation, cells were incubated at room temperature with Hoechst (1:800) for 15 min. For both assays, cells were then viewed using the INCell6000 analyzer with a 96-well plate adaptor. Images were collected with a 20X objective lens. Absolute numbers of cells were counted using Columbus Image Data Storage and Analysis System (PerkinElmer). Viability was measured as number of alive cells divided by total number of cells, both dead and alive, in a well. Proliferation was assessed by measuring the number of cells per well.

### Statistics and reproducibility

Statistical significance and *p* values were assessed by analysis of variance with Bonferroni correction and Student’s *t* tests. Error bars reflect SEM. Number of replicates and samples sizes are stated in the respective Figure Legend for each figure, with n value corresponding to independent experiments. All analysis was performed using R Statistical Software (v4.1.2; R Core Team 2021)^44^.

## Supplementary Information

Supplementary Information.

## Data Availability

The datasets generated and/or analysed during the current study are available in the Github repository, https://github.com/deannang/Ex-vivo-model-methods.
