# Supplementary material for: Investigating the mechanisms of peritoneal metastasis in gastric adenocarcinoma using a novel ex vivo peritoneal explant model
Source: Sci Rep. 2022 Jul 7;12:11499. doi: 10.1038/s41598-022-13948-x (PMC9262973; doi:10.1038/s41598-022-13948-x)
Supplement: Supplementary file 1 — Supplementary Information. [file 41598_2022_13948_MOESM1_ESM.pptx]

## Slide 1
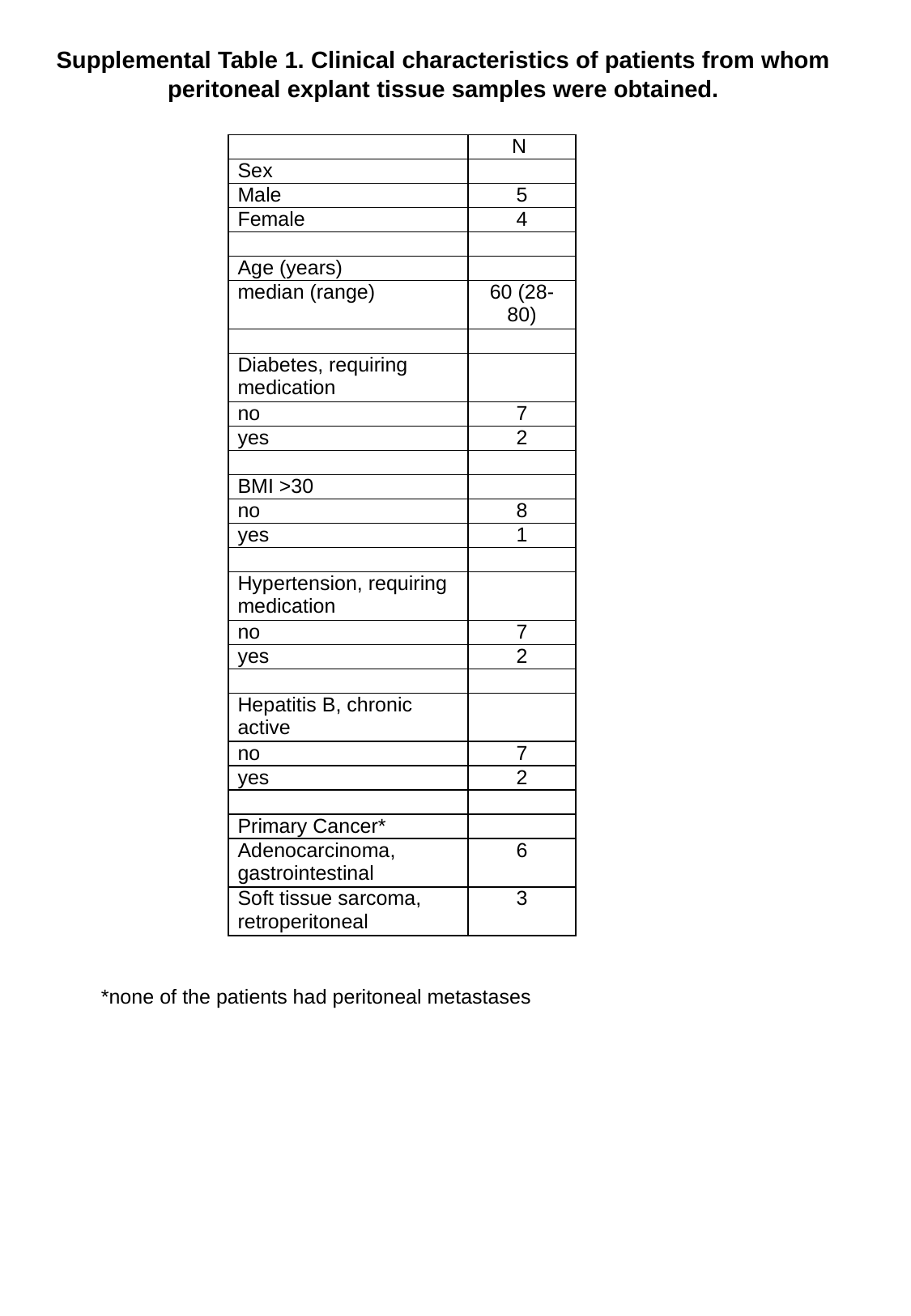

Supplemental Table 1. Clinical characteristics of patients from whom peritoneal explant tissue samples were obtained.
| | N |
| --- | --- |
| Sex | |
| Male | 5 |
| Female | 4 |
| | |
| Age (years) | |
| median (range) | 60 (28-80) |
| | |
| Diabetes, requiring medication | |
| no | 7 |
| yes | 2 |
| | |
| BMI >30 | |
| no | 8 |
| yes | 1 |
| | |
| Hypertension, requiring medication | |
| no | 7 |
| yes | 2 |
| | |
| Hepatitis B, chronic active | |
| no | 7 |
| yes | 2 |
| | |
| Primary Cancer\* | |
| Adenocarcinoma, gastrointestinal | 6 |
| Soft tissue sarcoma, retroperitoneal | 3 |
*none of the patients had peritoneal metastases

## Slide 2
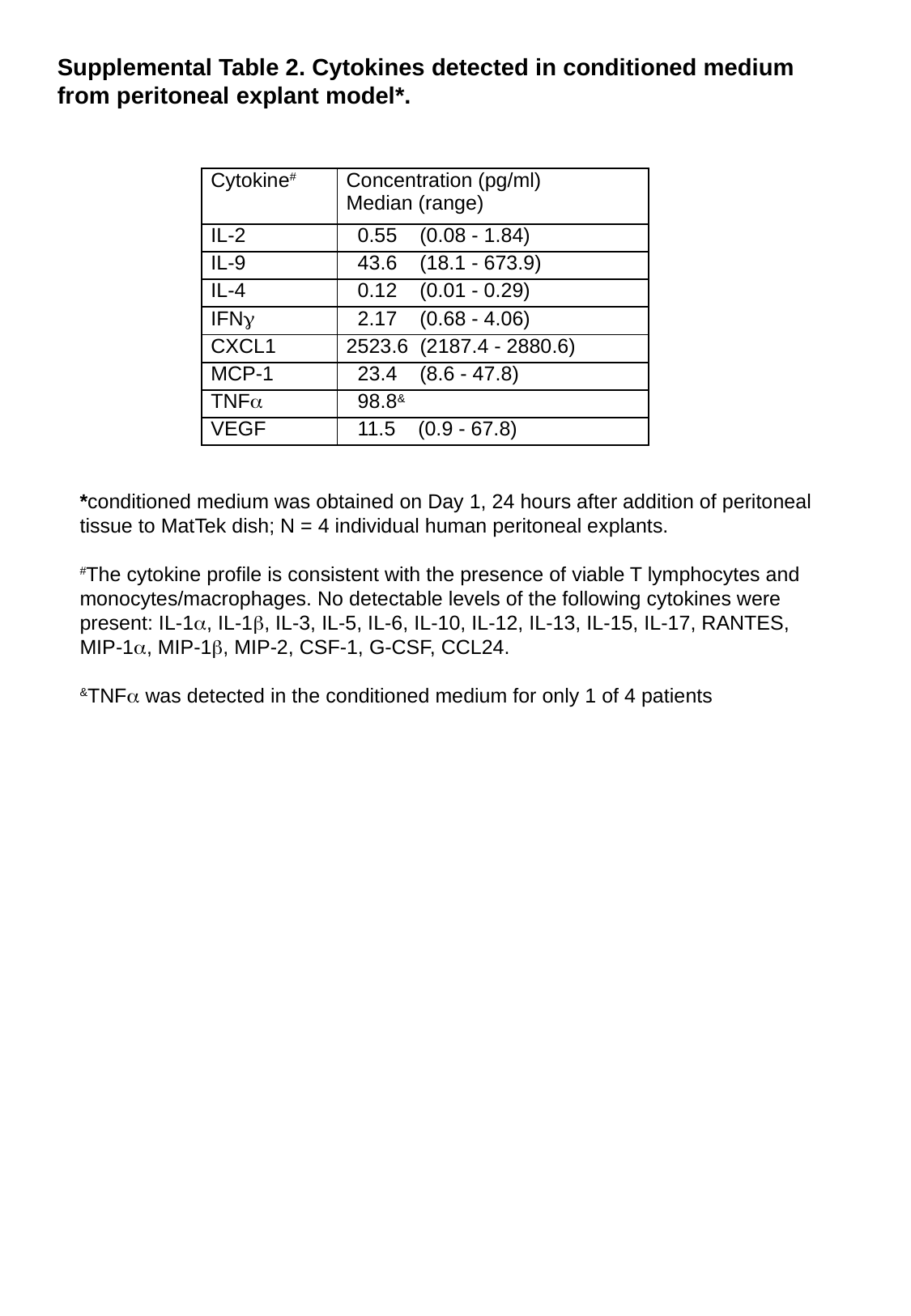

Supplemental Table 2. Cytokines detected in conditioned medium from peritoneal explant model*.
| Cytokine# | Concentration (pg/ml) Median (range) |
| --- | --- |
| IL-2 | 0.55 (0.08 - 1.84) |
| IL-9 | 43.6 (18.1 - 673.9) |
| IL-4 | 0.12 (0.01 - 0.29) |
| IFNγ | 2.17 (0.68 - 4.06) |
| CXCL1 | 2523.6 (2187.4 - 2880.6) |
| MCP-1 | 23.4 (8.6 - 47.8) |
| TNFα | 98.8& |
| VEGF | 11.5 (0.9 - 67.8) |
*conditioned medium was obtained on Day 1, 24 hours after addition of peritoneal tissue to MatTek dish; N = 4 individual human peritoneal explants.
#The cytokine profile is consistent with the presence of viable T lymphocytes and monocytes/macrophages. No detectable levels of the following cytokines were present: IL-1α, IL-1β, IL-3, IL-5, IL-6, IL-10, IL-12, IL-13, IL-15, IL-17, RANTES, MIP-1α, MIP-1β, MIP-2, CSF-1, G-CSF, CCL24.
&TNFα was detected in the conditioned medium for only 1 of 4 patients

## Slide 3
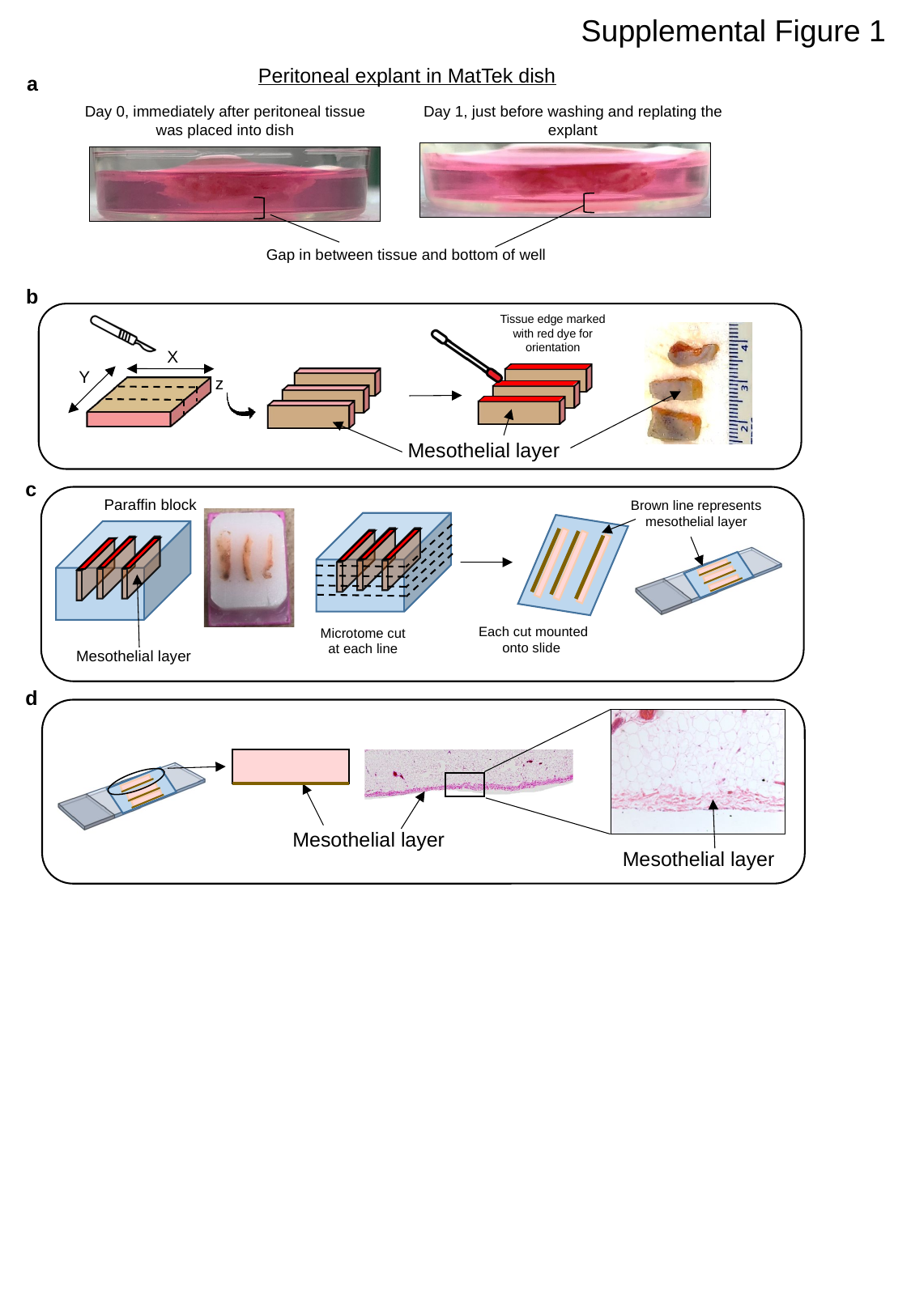

Supplemental Figure 1
Peritoneal explant in MatTek dish
a
Day 0, immediately after peritoneal tissue was placed into dish
Day 1, just before washing and replating the explant
Gap in between tissue and bottom of well
b
Tissue edge marked with red dye for orientation
X
Y
z
Mesothelial layer
c
Paraffin block
Brown line represents mesothelial layer
Each cut mounted onto slide
Microtome cut at each line
Mesothelial layer
d
Mesothelial layer
Mesothelial layer

## Slide 4
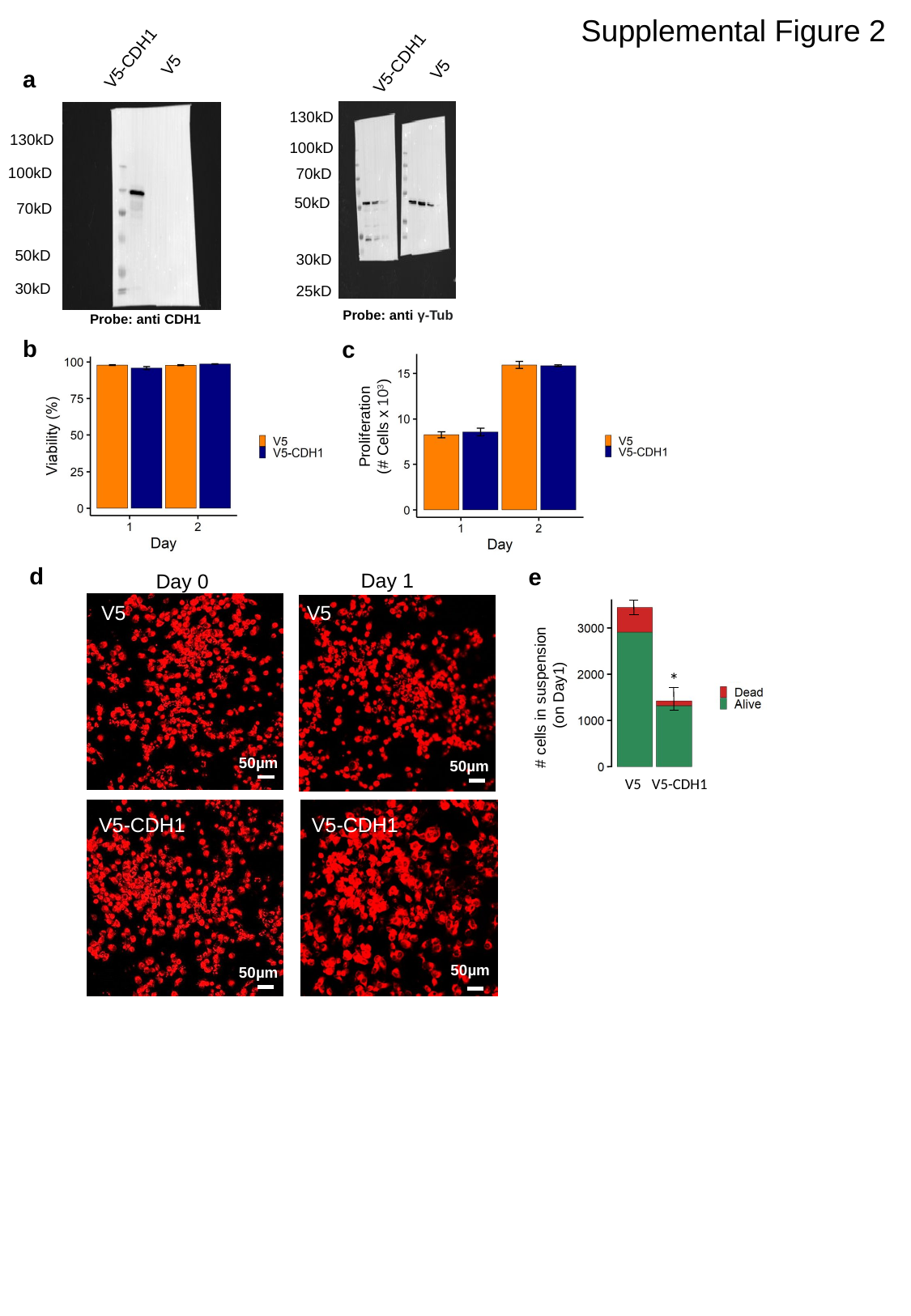

Supplemental Figure 2
V5-CDH1
V5-CDH1
V5
V5
a
130kD
130kD
100kD
100kD
70kD
50kD
70kD
50kD
30kD
30kD
25kD
Probe: anti γ-Tub
Probe: anti CDH1
b
c
Proliferation
(# Cells x 103)
d
e
Day 1
Day 0
*
# cells in suspension
(on Day1)
V5
V5
50µm
50µm
50µm
 V5 V5-CDH1
V5-CDH1
V5-CDH1
V5
50µm
50µm

## Slide 5
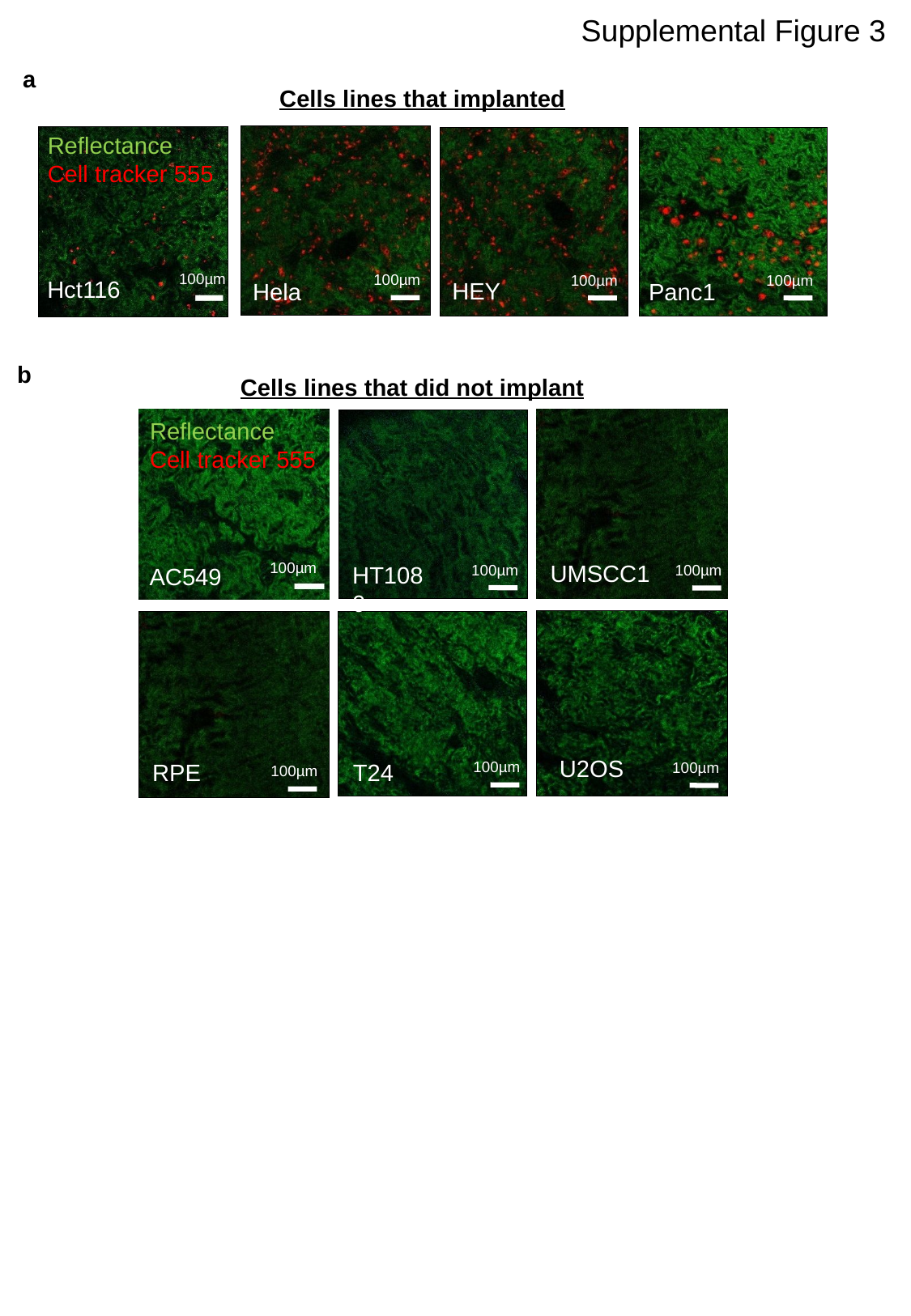

Supplemental Figure 3
a
Cells lines that implanted
Reflectance
Cell tracker 555
100µm
100µm
100µm
100µm
Hct116
HEY
Hela
Panc1
b
Cells lines that did not implant
Reflectance
Cell tracker 555
100µm
UMSCC1
100µm
100µm
HT1080
AC549
U2OS
RPE
100µm
T24
100µm
100µm

## Slide 6
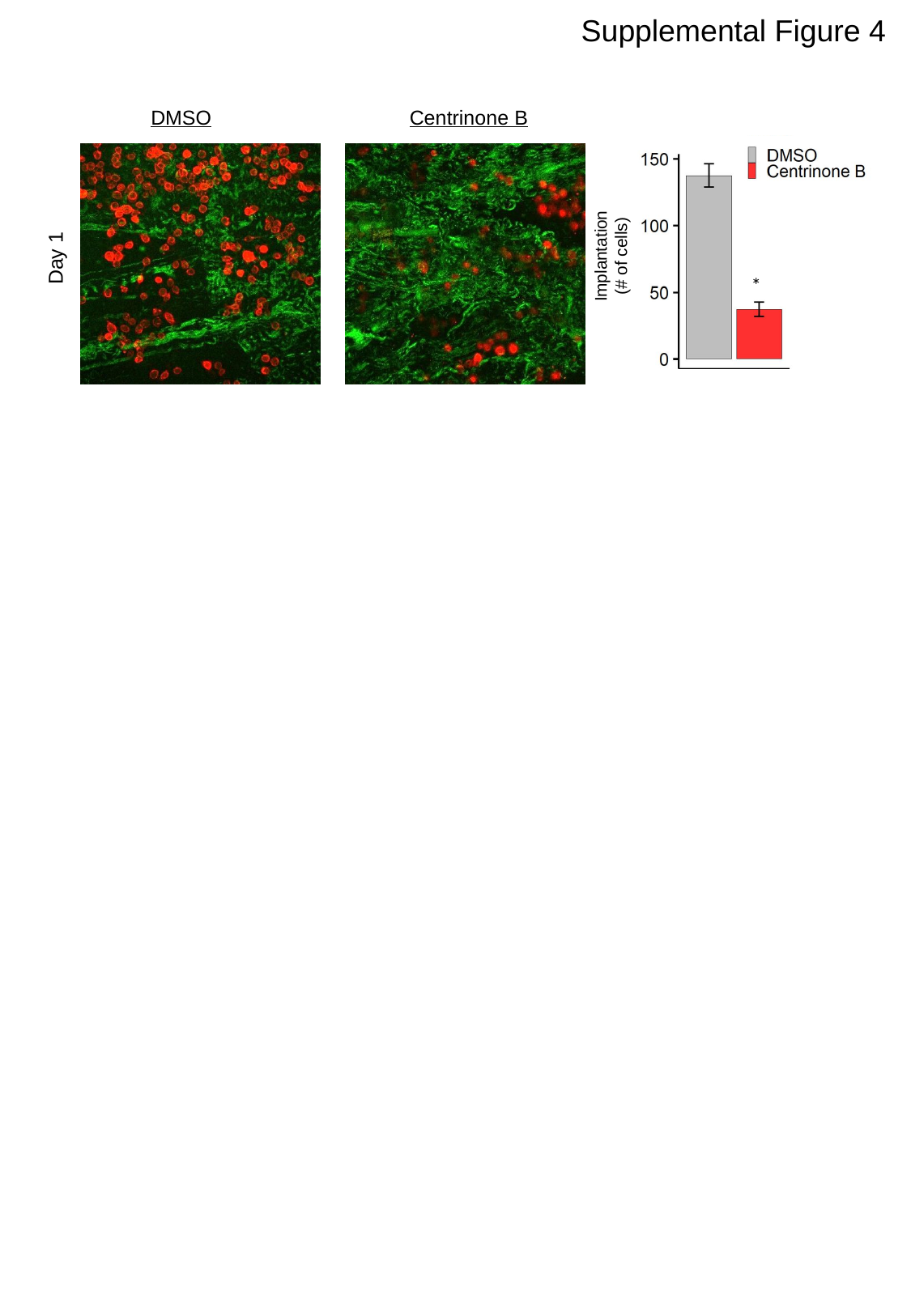

Supplemental Figure 4
DMSO
Centrinone B
Day 1
Implantation
(# of cells)
*

## Slide 7
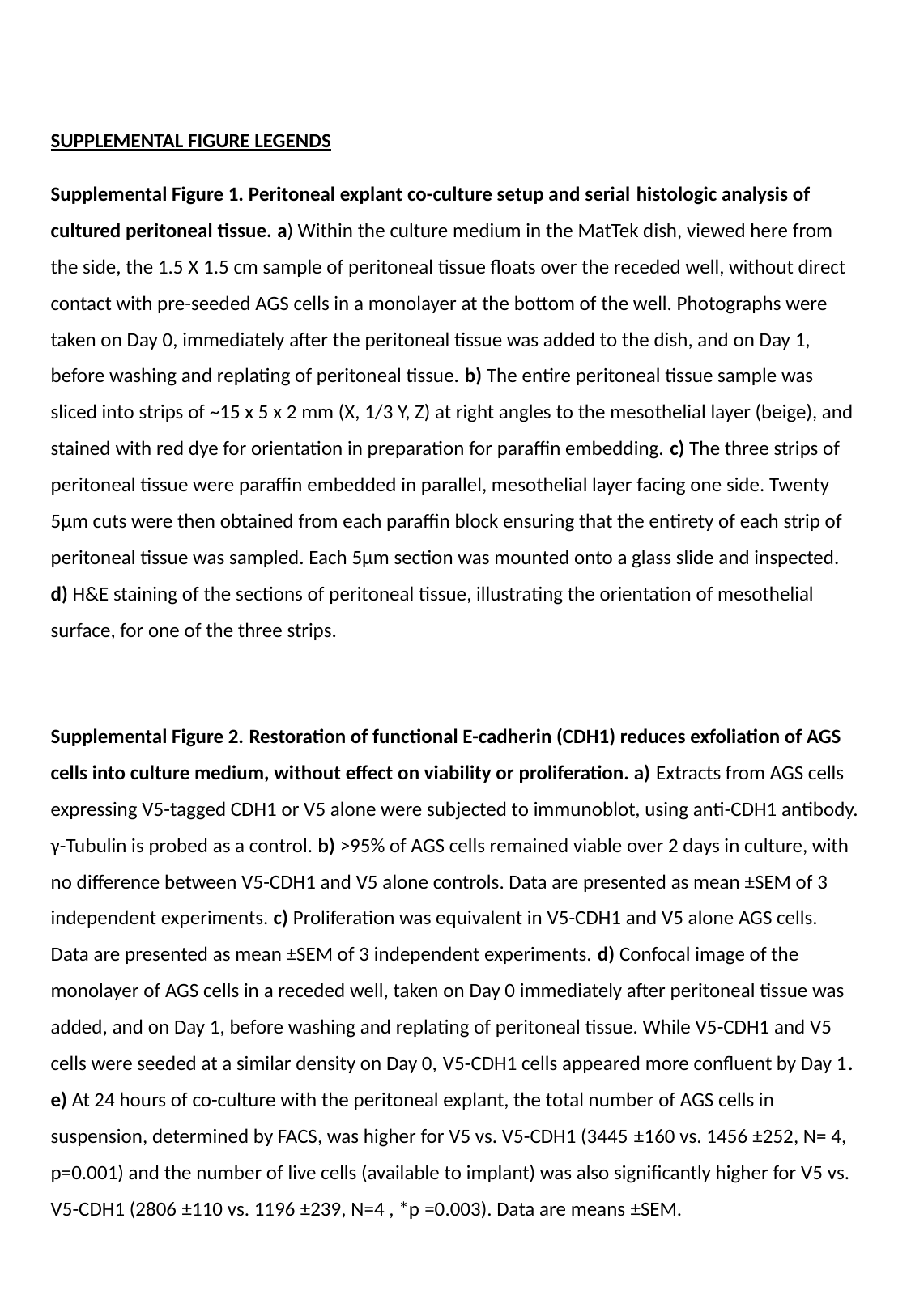

SUPPLEMENTAL FIGURE LEGENDS
Supplemental Figure 1. Peritoneal explant co-culture setup and serial histologic analysis of cultured peritoneal tissue. a) Within the culture medium in the MatTek dish, viewed here from the side, the 1.5 X 1.5 cm sample of peritoneal tissue floats over the receded well, without direct contact with pre-seeded AGS cells in a monolayer at the bottom of the well. Photographs were taken on Day 0, immediately after the peritoneal tissue was added to the dish, and on Day 1, before washing and replating of peritoneal tissue. b) The entire peritoneal tissue sample was sliced into strips of ~15 x 5 x 2 mm (X, 1/3 Y, Z) at right angles to the mesothelial layer (beige), and stained with red dye for orientation in preparation for paraffin embedding. c) The three strips of peritoneal tissue were paraffin embedded in parallel, mesothelial layer facing one side. Twenty 5µm cuts were then obtained from each paraffin block ensuring that the entirety of each strip of peritoneal tissue was sampled. Each 5µm section was mounted onto a glass slide and inspected. d) H&E staining of the sections of peritoneal tissue, illustrating the orientation of mesothelial surface, for one of the three strips.
Supplemental Figure 2. Restoration of functional E-cadherin (CDH1) reduces exfoliation of AGS cells into culture medium, without effect on viability or proliferation. a) Extracts from AGS cells expressing V5-tagged CDH1 or V5 alone were subjected to immunoblot, using anti-CDH1 antibody. γ-Tubulin is probed as a control. b) >95% of AGS cells remained viable over 2 days in culture, with no difference between V5-CDH1 and V5 alone controls. Data are presented as mean ±SEM of 3 independent experiments. c) Proliferation was equivalent in V5-CDH1 and V5 alone AGS cells. Data are presented as mean ±SEM of 3 independent experiments. d) Confocal image of the monolayer of AGS cells in a receded well, taken on Day 0 immediately after peritoneal tissue was added, and on Day 1, before washing and replating of peritoneal tissue. While V5-CDH1 and V5 cells were seeded at a similar density on Day 0, V5-CDH1 cells appeared more confluent by Day 1. e) At 24 hours of co-culture with the peritoneal explant, the total number of AGS cells in suspension, determined by FACS, was higher for V5 vs. V5-CDH1 (3445 ±160 vs. 1456 ±252, N= 4, p=0.001) and the number of live cells (available to implant) was also significantly higher for V5 vs. V5-CDH1 (2806 ±110 vs. 1196 ±239, N=4 , *p =0.003). Data are means ±SEM.

## Slide 8
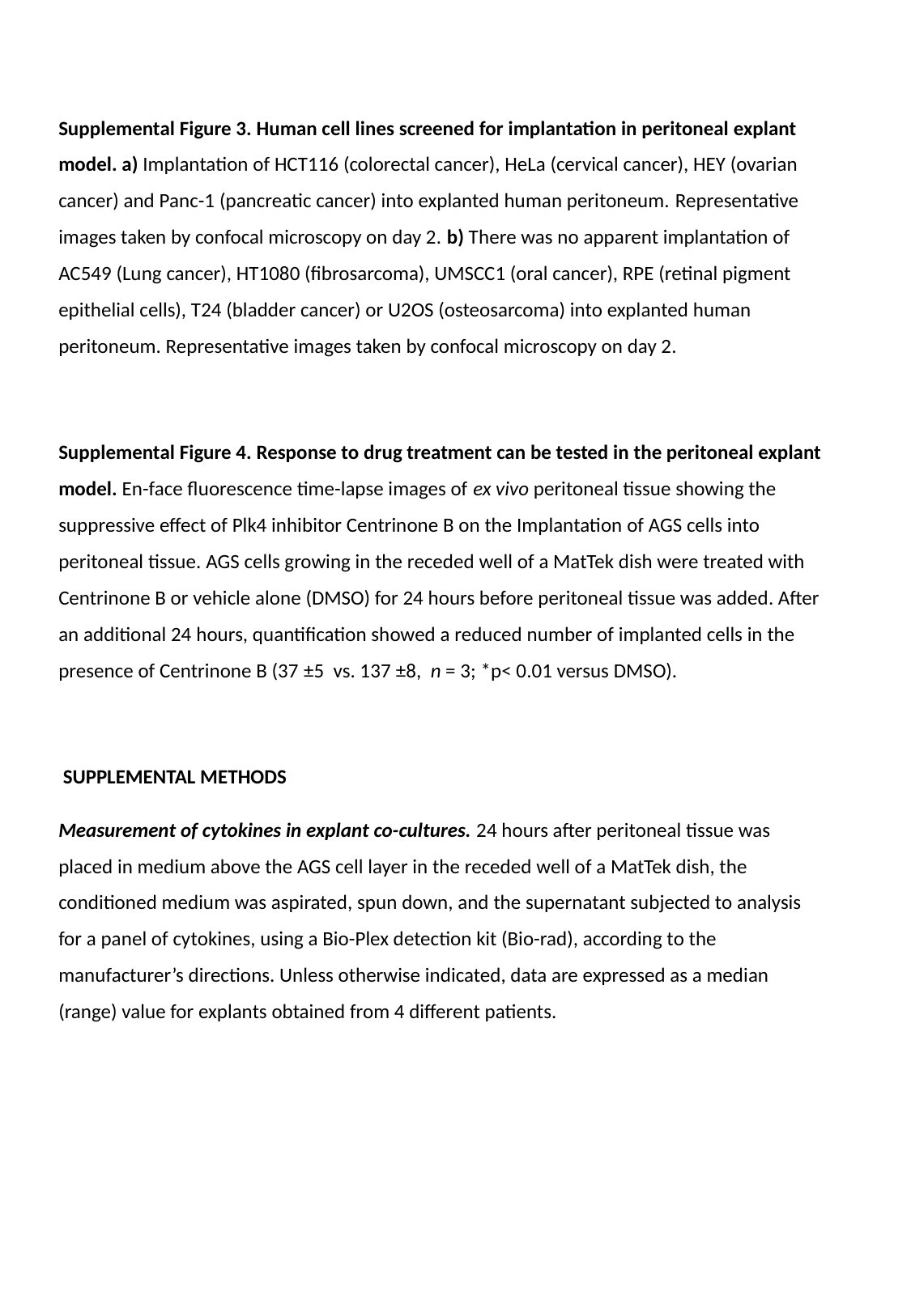

Supplemental Figure 3. Human cell lines screened for implantation in peritoneal explant model. a) Implantation of HCT116 (colorectal cancer), HeLa (cervical cancer), HEY (ovarian cancer) and Panc-1 (pancreatic cancer) into explanted human peritoneum. Representative images taken by confocal microscopy on day 2. b) There was no apparent implantation of AC549 (Lung cancer), HT1080 (fibrosarcoma), UMSCC1 (oral cancer), RPE (retinal pigment epithelial cells), T24 (bladder cancer) or U2OS (osteosarcoma) into explanted human peritoneum. Representative images taken by confocal microscopy on day 2.
Supplemental Figure 4. Response to drug treatment can be tested in the peritoneal explant model. En-face fluorescence time-lapse images of ex vivo peritoneal tissue showing the suppressive effect of Plk4 inhibitor Centrinone B on the Implantation of AGS cells into peritoneal tissue. AGS cells growing in the receded well of a MatTek dish were treated with Centrinone B or vehicle alone (DMSO) for 24 hours before peritoneal tissue was added. After an additional 24 hours, quantification showed a reduced number of implanted cells in the presence of Centrinone B (37 ±5 vs. 137 ±8,  n = 3; *p< 0.01 versus DMSO).
 SUPPLEMENTAL METHODS
Measurement of cytokines in explant co-cultures. 24 hours after peritoneal tissue was placed in medium above the AGS cell layer in the receded well of a MatTek dish, the conditioned medium was aspirated, spun down, and the supernatant subjected to analysis for a panel of cytokines, using a Bio-Plex detection kit (Bio-rad), according to the manufacturer’s directions. Unless otherwise indicated, data are expressed as a median (range) value for explants obtained from 4 different patients.
